# MYB induces the expression of the oncogenic corepressor SKI in acute myeloid leukemia

**DOI:** 10.18632/oncotarget.25051

**Published:** 2018-04-27

**Authors:** Miriam Frech, Sabine Teichler, Christine Feld, Caroline Bouchard, Hannah Berberich, Katharina Sorg, Marco Mernberger, Lars Bullinger, Uta-Maria Bauer, Andreas Neubauer

**Affiliations:** ^1^ Department of Internal Medicine and Hematology, Oncology and Immunology, Philipps University Marburg, Marburg 35033, Germany; ^2^ University Hospital Giessen and Marburg, Marburg 35033, Germany; ^3^ Institute of Molecular Biology and Tumor Research (IMT), School of Medicine, Philipps University Marburg, Marburg 35043, Germany; ^4^ Institute of Molecular Oncology, Philipps University Marburg, Marburg 35043, Germany; ^5^ Department of Internal Medicine III, University Hospital of Ulm, Ulm 89081, Germany

**Keywords:** oncogene, MYB, SKI, transcriptional regulation, acute myeloid leukemia

## Abstract

Acute myeloid leukemia (AML) arises through clonal expansion of transformed myeloid progenitor cells. The *SKI* proto-oncogene is highly upregulated in different solid tumors and leukemic cells, but little is known about its transcriptional regulation during leukemogenesis. MYB is an important hematopoietic transcription factor involved in proliferation as well as differentiation and upregulated in most human acute leukemias. Here, we find that MYB protein binds within the regulatory region of the *SKI* gene in AML cells. Reporter gene assays using MYB binding sites present in the *SKI* gene locus show MYB-dependent transcriptional activation. SiRNA-mediated depletion of *MYB* in leukemic cell lines reveals that MYB is crucial for *SKI* gene expression. Consistently, we observed a positive correlation of *MYB* and *SKI* expression in leukemic cell lines and in samples of AML patients. Moreover, *MYB* and *SKI* both were downregulated by treatment with histone deacetylase inhibitors. Strikingly, differentiation of AML cells induced by depletion of MYB is attenuated by overexpression of *SKI*. Our findings identify SKI as a novel MYB target gene, relevant for the MYB-induced differentiation block in leukemic cells.

## INTRODUCTION

Acute myeloid leukemia (AML) is characterized by the clonal expansion and arrest of differentiation of hematopoietic progenitor cells in the bone marrow. It accounts for 80% of acute leukemias [1]. AML patients have a 5-year survival rate of about 26% depending on the AML subtype and age of the patient [2]. AML is a genetically heterogenous disease, where chromosomal aberrations as well as point mutations in critical oncogenes act together during transformation [3, 4]. Frequently, the deregulation of important transcription factors, like CEBPA and RUNX1, as well as other proteins, like the phosphoprotein NPM or the class III receptor tyrosine kinase FLT3, is caused by chromosomal translocations or point mutations [5–11].

*MYB* was first described as a viral oncogene of avian leukemia viruses and is an important hematopoietic transcription factor involved in proliferation and differentiation of progenitor cells of the myeloid and lymphoid lineages [12–14]. Hence, *MYB* expression is precisely regulated during hematopoiesis, which is accomplished by various upstream transcription factors as well as microRNAs, for example microRNA(miR)-15a or miR193b-3p [15–17]. In many leukemic subtypes as well as solid tumors *MYB* is upregulated or mutated. Moreover, MYB transcriptional activity is highly dependent on its interaction with other transcription factors or co-factors [18]. On the one hand, the transactivation ability of MYB is inhibited by a corepressor complex containing TIF1beta, N-COR, mSIN3A and SKI, which further recruits histone deacetylase (HDAC) activity to MYB target genes [19]. On the other hand, MYB interacts with co-activators like MI2α, PRMT4, FLASH and CBP/p300 for transcriptional activation [20–23].

The oncoprotein SKI is an inhibitor of TGFβ signaling by binding to the SMAD2/3/4 complex [24–27]. However, it acts not only as a transcriptional co-repressor but also as a transcriptional co-activator, e.g. for NFI and FHL2 [28, 29]. Besides its normal cellular role *SKI* is upregulated in different solid tumors and leukemias [30]. Our group showed that SKI contributes to the differentiation block in AML by co-repressing the activity of the hematopoietic transcription factor RARα, which can partially be reversed upon treatment with the HDAC inhibitor (HDACi) valproic acid (VPA) [31]. High *SKI* expression was further suggested to inhibit therapy responses of AML patients treated with chemotherapy combined with all-trans retinoic acid (ATRA) [32]. Moreover, *SKI* is upregulated in AML, especially with monosomy 7 or deletion 7q (-7/del7q) [31]. We identified *SKI* as a target of miR-29a, which is encoded on chromosome 7q. In AML with -7/del7q, miR-29a expression is downregulated leading to increased *SKI* expression [33]. Although SKI function is well characterized, the transcriptional regulation of the *SKI* gene itself is still enigmatic, in particular in leukemia and other tumor entities with high *SKI* expression levels but no chromosome 7 deletion. Here, we provide insights in the transcriptional regulation of the human *SKI* gene by the transcription factor MYB and identify *SKI* as an important downstream target of the oncogenic function of MYB.

## RESULTS

### *SKI* regulatory region contains four putative MYB consensus sites

**Figure 1:**
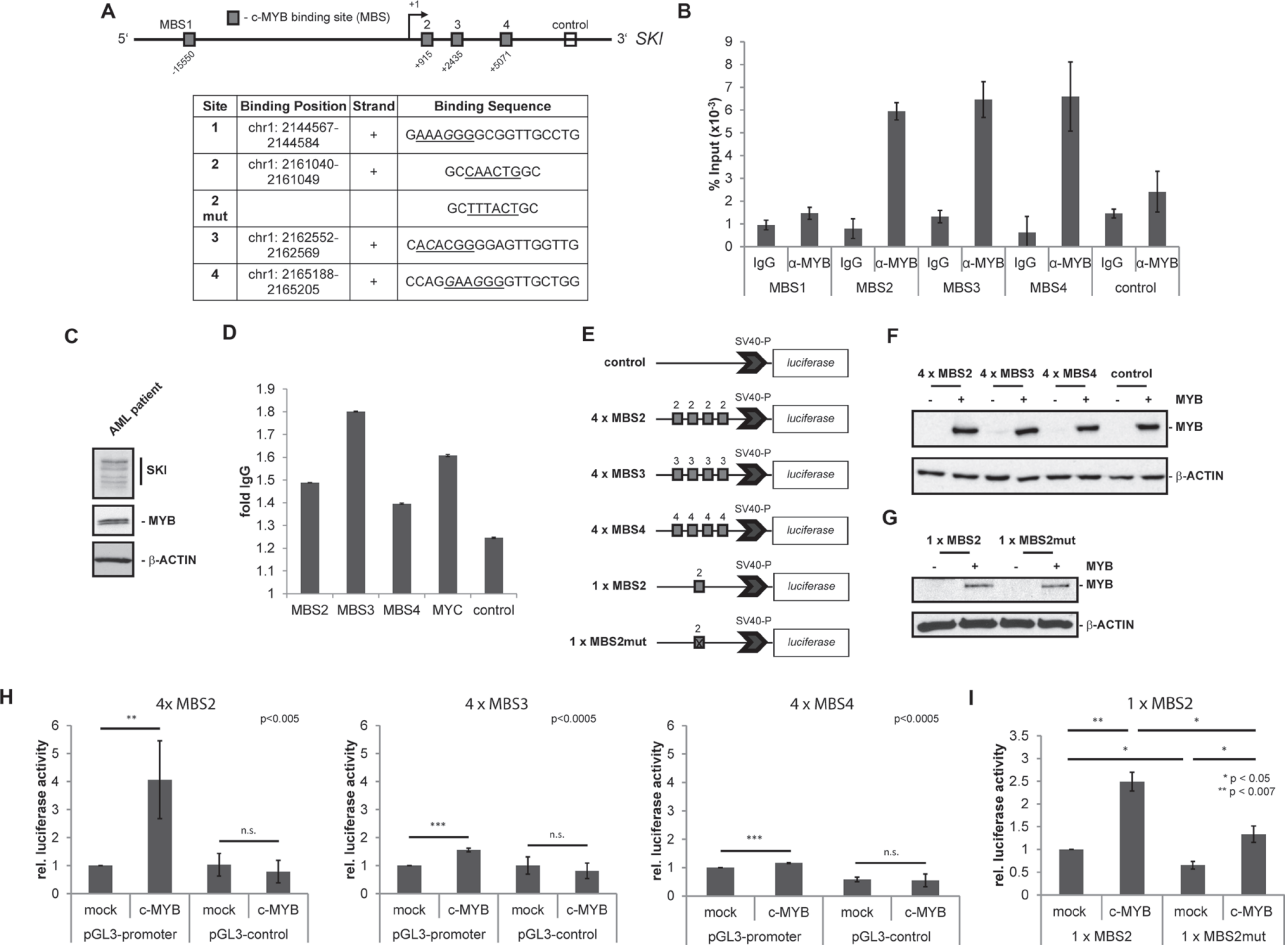
MYB binds to the regulatory region of the human *SKI* gene and induces *SKI* expression (**A–D**) Binding of MYB to its putative DNA binding sites in the human *SKI* regulatory region. (A) Scheme of *SKI* gene and its regulatory regions with four putative MYB DNA binding sites (MBS1 - MBS4, grey boxes). The open box marks the downstream control region used for ChIP/qPCR. The +1 marks the transcriptional start site of *SKI*. The table depicts positions and sequences of the MBS with the MYB consensus motif (underlined) and the mutated consensus motif of MBS2. Hg38 (GRCh38) was used as human reference genome. (B) ChIP/qPCR analysis of MYB binding to the MYB DNA binding sites of the SKI regulatory region in HL60 cells. Data are shown as percent of amplified product relative to input. The downstream control region (control) depicts the interaction with an unrelated region as a negative control. Data are representative of three independent experiments performed in triplicate (mean ± s.d.). Isotype-specific IgG served as control antibody. (C) Western Blot analysis showing endogenous expression of MYB and SKI in the AML patient cells used in (D). β-ACTIN served as loading control. (D) ChIP/qPCR analysis of MYB binding to the MYB DNA binding sites of the SKI regulatory region in AML patient cells. Data are shown as relative fold to IgG. The downstream control region (ctr) served as a negative control and MYC served as a positive control. Data are representative of two independent experiments performed in triplicate (mean ± s.d.). (**E**–**I**) MBS2, MBS3 and MBS4 luciferase assays ± *MYB* overexpression. (E) pGL3-promoter constructs containing four consecutive consensus sites of MBS2, MBS3, MBS4 or a single consensus site of MBS2 unmutated or mutated or pGL3-control without MBS as negative control. (F–G) Representative Western Blot analyses showing *MYB-HA* overexpression in HEK293T cells co-transfected with MYB-HA or empty vector (mock), pRL-TK and pGL3-promoter-4 × MBS2, -4 × MBS3, -4 × MBS4 or pGL3-control (F) or pGL3-promoter-1 × MBS2, -1 × MBS2mut (G). (**H**) HEK293T were co-transfected with MYB-HA or empty vector (mock), pGL3-promoter-4 × MBS2, -4 × MBS3, -4 × MBS4 or pGL3-control and pRL-TK as an internal standard. The data (*n* = 4, mean ± s.d.) were normalized to *renilla* luciferase activity. pGL3-promoter constructs with MBS co-transfected with the empty vector (mock) were set to 1. ^**^P < 0.005, ^***^P < 0.0005. (I) HEK293T were co-transfected with MYB-HA or empty vector (mock), pGL3-promoter-1 × MBS2 or -1 × MBS2mut. The data (*n* = 3, mean ± s.d.) were normalized to total protein concentration. Construct pGL3-promoter-1 × MBS2 co-transfected with the empty vector (mock) was set to 1. ^*^P < 0.05, ^**^P < 0.007.

Since little is known about transcriptional regulation of *SKI* oncogene, we performed *in silico* analysis of the *SKI* regulatory region for putative transcription factor binding sites using the Champion ChiP Transcription Factor Search Portal (QIAGEN). The analysis revealed four putative DNA binding sites, designated MBS1-MBS4, corresponding to the consensus site A/CAACG/TG of the hematopoietic transcription factor MYB in the upstream regulatory and transcribed region of *SKI* (Figure 1A). The MYB binding site MBS2 thereby shows the highest agreement with the consensus sequence (Figure 1A, table underlined).

### MYB induces *SKI* expression by binding directly to the regulatory region of the *SKI* gene

In order to study the interaction of the transcription factor MYB with its putative binding sites in the *SKI* regulatory region, we performed ChIP-qPCR experiments with an antibody against MYB in the AML cell line HL60 that endogenously expresses *MYB* and *SKI*. We used specific flanking primers for amplifying the MYB binding sites MBS1, MBS2, MBS3 and MBS4. As control we amplified an unrelated, further downstream region in the *SKI* gene, in which no MYB binding sites were predicted (Figure 1A). The results showed a binding of MYB to the predicted binding sites MBS2, MBS3 and MBS4, but not to MBS1 and the downstream control region (Figure 1B). To validate these findings *in vivo*, we performed ChIP-qPCR analyses on primary material from an AML patient endogenously expressing MYB and SKI (Figure 1C, Supplementary Table 3). Here, we also found MYB to be enriched at the predicted MYB binding sites MBS2, MBS3 and MBS4 but not the unrelated downstream control region (Figure 1D). Enrichment of MYB at its known target gene *MYC* was added as a positive control (Figure 1D). Hence, MYB seems to interact also in primary AML patient cells with the predicted sites in the SKI regulatory region. Further analyses of a published and online available MYB ChIP DNA-sequencing (ChIP-seq) dataset from the human lymphoid cell line Jurkat [34] also revealed an enrichment of MYB in the *SKI* regulatory region, upstream of the *SKI* transcriptional start site and at an internal gene region (Supplementary Figure 1A).

To investigate whether MYB could influence *SKI* gene transcription via MYB binding sites MBS2, MBS3 and MBS4, we performed dual-luciferase reporter gene assays in HEK293T cells. Therefore, cells were transfected with constructs containing the luciferase reporter gene under control of the SV40 promoter with four copies each of the MYB consensus sites MBS2, MBS3, MBS4 or with one copy of MBS2 unmutated or mutated (Figure 1E). A construct containing the luciferase reporter gene under control of the SV40 promoter without MYB consensus sites served as an additional negative control (Figure 1E). Subsequently, the luciferase activity was measured in the absence or presence of *MYB* overexpression. In this regard, MYB overexpression was confirmed by Western Blot analyses (Figure 1F, 1G). Upon *MYB* overexpression the promoter activity of the 4 x MBS2-containing reporter was induced 4-fold compared to mock-transfected cells (Figure 1H). The *MYB* overexpression resulted in a minor increase of the promoter activity of MBS3- and MBS4-containing reporters and no increase in case of the control constructs (Figure 1H). Additionally, cells transfected with the control vector without MYB binding sites showed no promoter induction upon MYB overexpression (Figure 1H). Further luciferase reporter gene assays were executed, in which the constructs contained only a single intact MBS2 (1 x MBS2) or a single mutated MBS2 (1 x MBS2mut) (Figure 1E). The promoter activity of the 1 x MBS2-containing reporter was induced 2.5-fold upon MYB overexpression compared to mock-transfected cells (Figure 1I, left). However, mutation of the MBS2 led to destruction of the site and a significant decrease in the promoter activity upon MYB overexpression (Figure 1I, right). Similar results were obtained with the constructs 4 x MBS2 and 4 x MBS2mut (Supplementary Figure 1B). Thus, MYB binds directly to predicted sites in the regulatory region of the *SKI* gene and induces *SKI* expression mainly via the MYB consensus site MBS2.

### Downregulation of *MYB* results in a reduced *SKI* expression in AML cell lines

**Figure 2:**
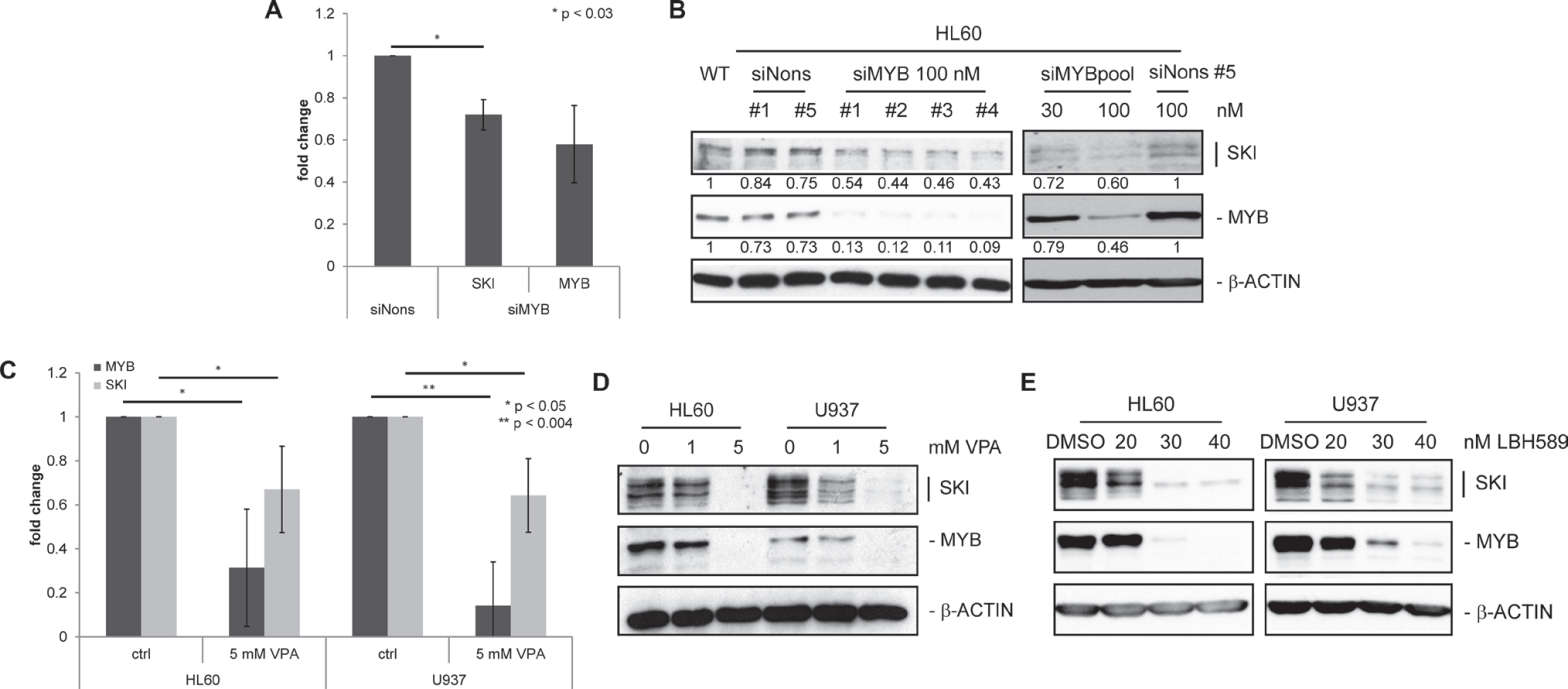
*SKI* expression is decreased upon down-regulation of *MYB* in AML cell lines (**A**) RT-qPCR analysis of three independent experiments performed in duplicates for *MYB* and *SKI* transcripts in HL60 transfected with siMYB #1 (siMYB) or siNonsense #5 (siNons). Cells were harvested 24 h after transfection. Values are normalized to *GAPDH* and plotted relative to siNons (mean ± s.d.). ^*^P < 0.03. (**B**) Western Blot analysis for SKI and MYB in HL60 transfected with indicated amounts of siMyb #1-4 or pool (mixed #1–4) or siNons #1or #5. Cells were harvested 24 h after transfection. β-ACTIN served as loading control. Numbers indicate relative mean values INT of quantified SKI and MYB protein bands normalized to quantified β-ACTIN bands. (**C**) RT-qPCR analysis of four independent experiments performed in duplicates for *MYB* and *SKI* transcripts in HL60 and U937 cells treated with 0 (ctrl) or 5 mM VPA for 48 h. Values are normalized to *GAPDH* and plotted relative to ctrl cells (mean ± s.d.). ^*^P < 0.05, ^**^P < 0.004. (**D**, **E**) Western Blot analysis for SKI and MYB in AML cell lines HL60 and U937 treated with 0, 1 or 5 mM VPA (D) or DMSO (solvent control), 20, 30 or 40 nM LBH589 (E) for 48 h. β-ACTIN served as loading control.

To further investigate the function of MYB in activating *SKI* transcription, we transfected HL60 AML cells showing high MYB and SKI protein levels with siRNAs targeting *MYB* or control siRNAs and analyzed *SKI* expression on RNA and protein levels. As shown in Figure 2A, a knockdown of *MYB* resulted in a significant reduction of *SKI* transcript levels. Accordingly, SKI protein levels were reduced upon knockdown of *MYB* with four different siRNAs in HL60 cells or with the pool of all four siRNAs in U937 cells (Figure 2B, left panel; Supplementary Figure 2A). This protein reduction was dose-dependent, as shown by transfection of HL60 cells with increasing doses of siMYB (Figure 2B, right panel). In all cases transfection with siRNAs had no effect on the protein levels of the internal control (Figure 2B and Supplementary Figure 2A, β-ACTIN). Furthermore, we show that decreased *SKI* expression was secondary to a decrease in *MYB* expression in RNAi-mediated knockdown experiments in HL60 cells, additionally excluding off-target effects (Supplementary Figure 2B). Vice versa, MYB overexpression in K562 cells, a cell line with low MYB and SKI protein levels, induced SKI expression significantly (Supplementary Figure 2C). These data further support the notion that MYB activates *SKI* gene transcription.

The histone deacetylase inhibitor (HDACi) valproic acid (VPA) is known to reduce MYB protein levels in a neural tube defect mouse model [35]. Furthermore VPA attenuates the repressive effect of SKI on differentiation in AML [31]. To further analyze the impact of HDACi on the expression levels of MYB and SKI, we treated the AML cell lines HL60 and U937, both endogenously expressing *MYB* and *SKI*, with VPA and the more potent HDACi LBH589. RT-qPCR and Western Blot analyses showed a concordant decrease of *MYB* and *SKI* levels upon VPA treatment (Figure 2C and 2D). Likewise, treatment with LBH589 induced a concordant decrease of both proteins (Figure 2E).

### *MYB* and *SKI* expression highly correlate in several leukemia cell lines and primary AML patient cells

**Figure 3:**
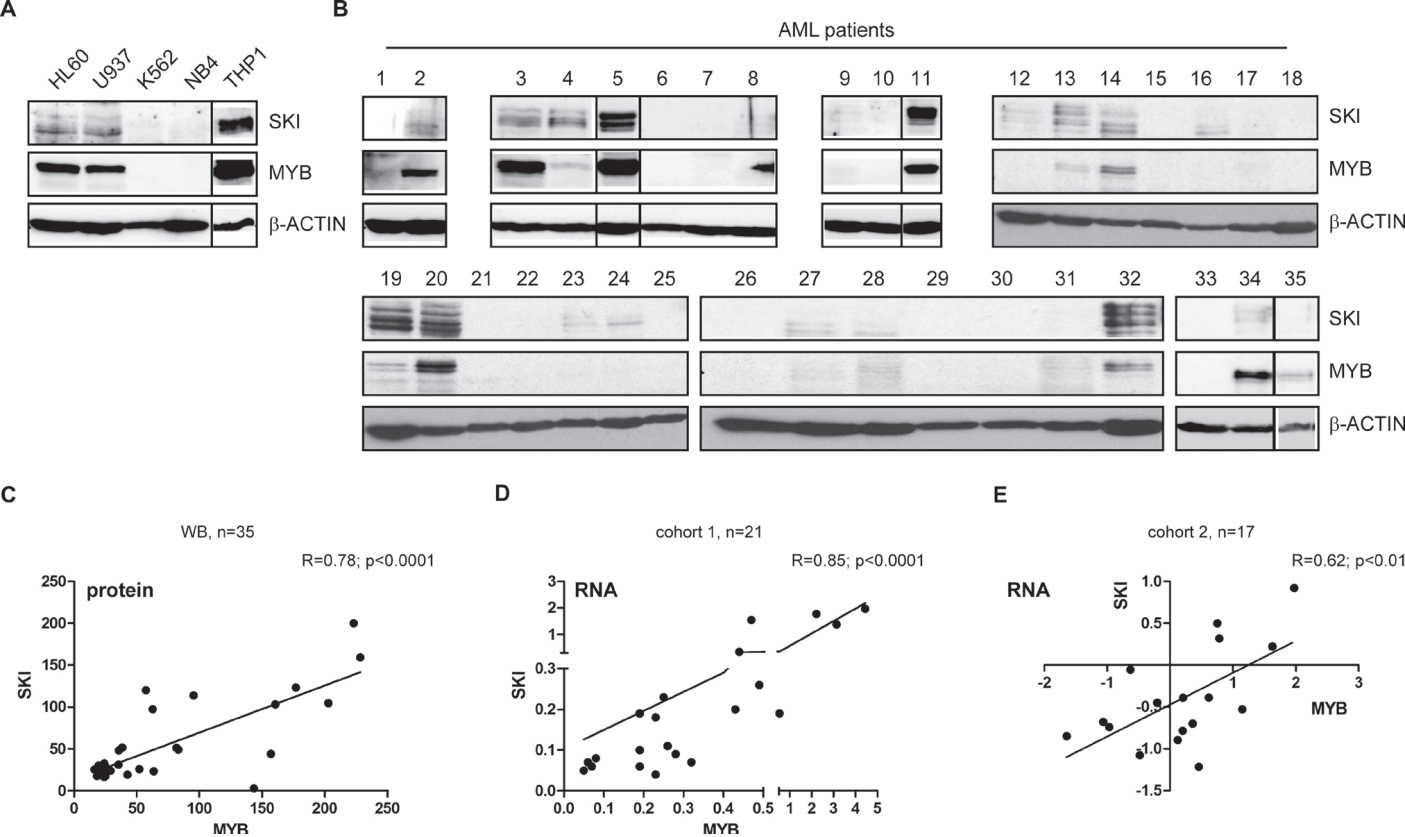
*MYB* and *SKI* expression highly correlate in AML cell lines and primary hematopoietic cells of AML patients Western Blot for SKI and MYB in AML cell lines HL60, U937, THP1, NB4 and CML cell line K562 (**A**) or in AML patients (*n* = 35) (**B**). β-ACTIN served as loading control. (**C**) Scatter plot depicts the correlation of *MYB* and *SKI* protein levels determined by band quantification of the Western Blots of AML patients (*n* = 35) shown in (b). Pearson's correlation: R = 0.78, *P <* 0.0001. (**D**) Scatter plot depicts the correlation of *MYB* and *SKI* mRNA levels in samples of mononuclear cells of AML patients (*n* = 21, cohort 1) (RT-qPCR data). Data of *MYB* and *SKI* were normalized to *GAPDH* and shown relative to the Kasumi-1 cell line. Pearson's correlation: R = 0.85, *P <* 0.0001. (**E**) Scatter plot depicts the correlation of *MYB* and *SKI* mRNA levels in samples of untreated AML patients (*n* = 17, cohort 2) (cDNA array data) [36]. Pearson's correlation: R = 0.62, *P <* 0.01.

In order to examine if *MYB* and *SKI* expression might correlate in AML cell lines we analyzed MYB and SKI protein levels in HL60, U937, NB4 and THP1 cells as well as in the CML cell line K562 (Figure 3A). In these five cell lines MYB and SKI protein expression levels revealed a similar pattern. MYB and SKI levels were high in HL60, U937 and THP1 cells, whereas both proteins were expressed at low abundancy in K562 and NB4 cells. Interestingly, SKI and MYB expression inversely correlated with the occurrence of the translocations t(9;22)(*BCR-ABL*) and t(15;17)(*PML-RARα*) in K562 and NB4 cells, respectively.

Subsequently, we aimed to study the relevance of these observations in primary AML cells. Hence, we determined MYB and SKI protein levels of 35 different AML patients via Western Blot analysis (Figure 3B, Supplementary Table 1). Quantification of the MYB and SKI protein bands revealed a significant positive correlation of MYB and SKI protein levels *in vivo* (Figure 3C), meaning detectable MYB expression coincided with the presence of SKI protein. Here, MYB and SKI expression seems not to be correlated with any specific underlying genetic aberration (*cf*. Supplementary Table 1). To validate our findings, we analyzed *MYB* and *SKI* transcript levels in two cohorts of primary AML patient cells. In cohort 1 *MYB* and *SKI* gene expression of 21 AML patients was examined via RT-qPCR (Supplementary Table 2). As depicted in Figure 3D, the samples showed a highly significant positive correlation between *MYB* and *SKI* expression (Pearson's correlation: R = 0.85; *P* < 0.0001). Cohort 2 consists of cDNA microarray data of 17 samples of untreated AML patients collected within the trials AMLSG 07-04 and AMLSG 06-04 (GEO accession no. GSE32240 [36]). Similarly, the expression data of this cohort showed a significant positive correlation between *MYB* and *SKI* expression levels (Figure 3E, Pearson's correlation: R = 0.62; *P* < 0.01).

### *SKI* overexpression reduces MYB dependent differentiation of AML cells

**Figure 4:**
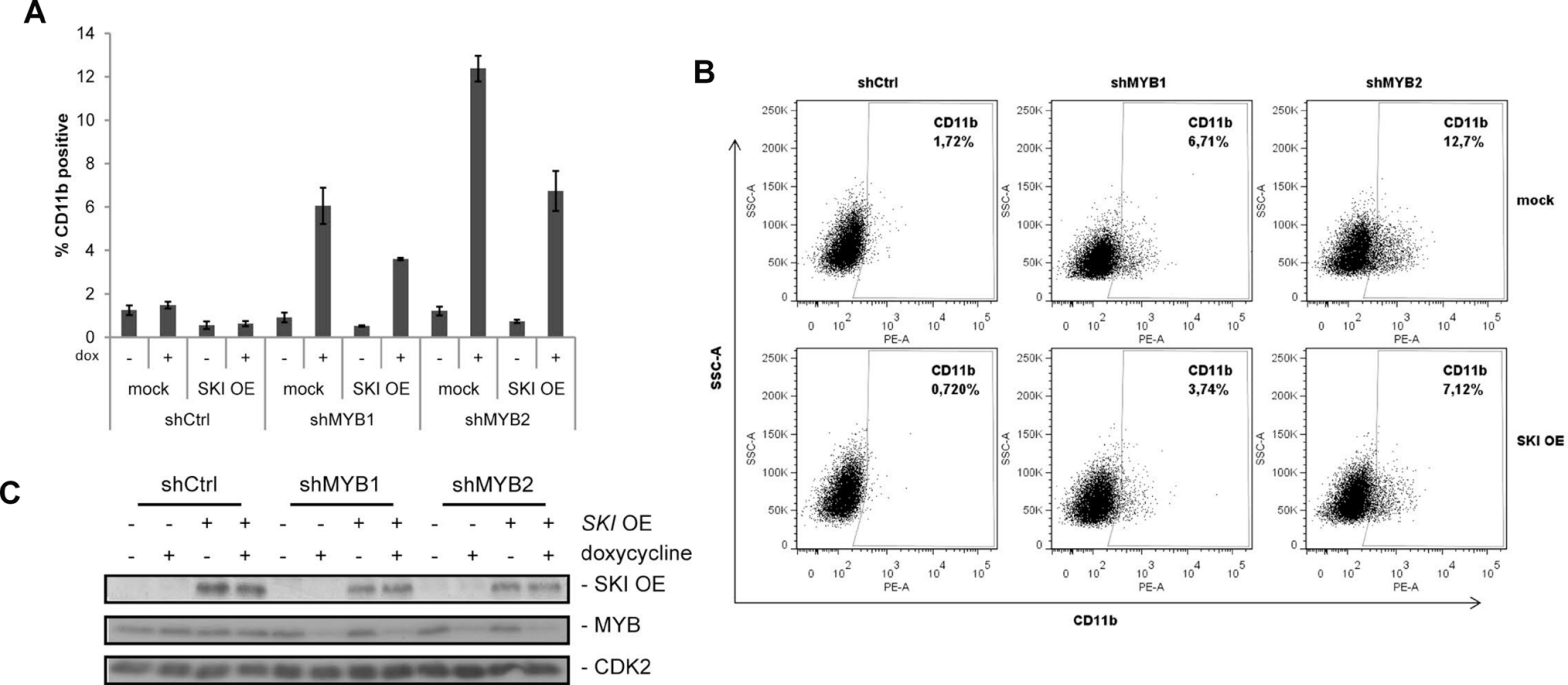
*SKI* overexpression attenuates MYB dependent differentiation in AML cells (**A**) Expression of CD11b was analyzed by FACS in HL60 expressing alternative shRNAs (shMYB1, shMYB2) targeting *MYB* or a control shRNA (shCtrl) in absence or presence of *SKI* overexpression (SKI OE). The empty vector (mock) served as negative control for *SKI* overexpression. Expression of shRNAs was induced by doxycycline (dox) and cells were measured via FACS (anti-human CD11b-PE) 48 h later. Bar graphs show mean values ± s.d. of % CD11b-positive HL60 of a representative experiment performed in technical triplicates from four independent experiments with similar results. (**B**) Data with doxycycline induction are shown as one typical result from four independent experiments with similar results. The experiment was performed and measured as described in (A). (**C**) Representative Western Blot analyses of the experiments performed in (A) and (B) showing *SKI* overexpression and MYB knockdown by shRNAs in the doxycycline-inducible HL60 cells.

Finally, we addressed the question of whether *SKI* is a relevant downstream target of the oncogenic functions of MYB. For this reason, we generated HL60 cell lines stably expressing either shRNAs targeting *MYB* (shMYB) or a control shRNA (shCtrl) in a doxycycline-inducible manner. Additionally, these cells were retrovirally infected for overexpression of *SKI* or as control without *SKI* overexpression (mock). Transduced cells were stimulated with or without doxycycline and myeloid cell differentiation was measured after 48 h by monitoring the differentiation marker CD11b and cell size via flow cytometry. Besides expression of differentiation markers like CD11b, HL60 cells undergo morphological changes during differentiation including decrease in cell size [37, 38]. As expected, *MYB* knockdown alone (mock cells) induced with both alternative shRNAs (shMYB1 and shMYB2) caused an increase in expression of the differentiation marker CD11b compared to cells without doxycycline treatment as well as shCtrl transduced cells (Figure 4A and 4B, mock). Notably, *SKI* overexpression reduced the MYB-dependent differentiation and led to a decreased expression of *CD11b* (Figure 4A, 4B). This effect was shown to be significant by means of the experiments engaging shMYB2 (Supplementary Figure 3A). As expected, increase or decrease of HL60 differentiation was also accompanied by decrease or increase in cell size, respectively (Supplementary Figure 3B, FSC-A). *MYB* knockdown upon doxycycline stimulation and *SKI* overexpression were confirmed by Western Blot analysis (Figure 4C). Here, MYB knockdown also showed concomitant reduction of endogenous SKI protein levels (Supplementary Figure 3C) confirming the results of the siMYB knockdown experiments (Figure 2B). These data show that SKI contributes to the differentiation block of AML cells in a MYB-dependent manner.

Taken together, these data reveal that the hematopoietic transcription factor MYB binds to the regulatory region of *SKI* proto-oncogene and activates its expression *in vitro* in AML cell lines. Moreover, there is a highly significant positive correlation of *MYB* and *SKI* expression *in vivo* in AML patient cells. We further identify *SKI* as a relevant downstream target gene of MYB that contributes to the differentiation blocking activity of MYB and seems to belong to the oncogenic transcriptional response caused by MYB in AML cells.

## DISCUSSION

In this study, we provide evidence that in myeloid cells the transcription factor MYB binds to transcribed regulatory regions of the proto-oncogene *SKI* MBS2, MBS3 and MBS4. However, in lymphoid cells MYB also interacts with *SKI* regulatory regions, but these seem to differ from the myeloid regions, as shown by the ChIP-seq analysis of Jurkat cells [34]. In this regard, Bengtsen *et al.* [39] have reported before that MYB transcriptional activity is highly cell type-dependent, which can also be due to different interaction sites in the target genes. Moreover, in our reporter gene assays, overexpression of *MYB* led to luciferase reporter gene induction in the presence of MYB myeloid consensus site MBS2, which originates from the regulatory regions of the *SKI* gene. Upon destruction of this site by mutation, the induction was significantly decreased. Furthermore, upon MYB overexpression SKI expression was significantly induced in K562, a leukemic cell line showing low endogenous MYB and SKI protein levels. In the reporter gene assays induction via MBS2 only may be due to the fact that this binding site shows the highest agreement with the predicted MYB consensus site and that experiments were performed in the non-myeloid HEK293T system. Vice versa *MYB* knockdown in AML cells resulted in decreased *SKI* RNA and protein levels *in vitro*. Moreover, treatment with the HDACi VPA and LBH589 caused a comparable and dose-dependent decrease of MYB and SKI protein levels, which may have a therapeutic impact for AML patients with both increased MYB and SKI levels. In different AML cell lines MYB and SKI protein expression levels were highly positively correlated. This is in agreement with results from different cohorts of AML patient samples, where transcript and protein levels of *MYB* and *SKI* were significantly positively correlated. Finally, we show that the MYB-dependent differentiation block in AML cells is in part executed by its target gene *SKI*, as myeloid differentiation induced by *MYB* knockdown was reduced by *SKI* overexpression.

These data raise the question by which mechanism and to which extent SKI might account for MYB's cellular function in normal and transformed cells and *vice versa*. Our data indicate that both proteins cooperate in transformed cells. However, whether they act in concert also under physiological conditions and which cellular state this might be, has so far not been answered. *MYB* and *SKI* are both predominantly expressed in immature hematopoietic stem cells [40–45]. Nevertheless, regulation of *SKI* expression in different cell types and during normal hematopoiesis could involve MYB but also additionally or exclusively other transcription factors. Consequently, different and non-correlative expression levels of *MYB* and *SKI* might occur during the normal regulation of stem cells as well as progenitor cells and differentiation into myeloid cells. MYB activity and its selection of target genes strongly depends on the interaction with other transcription factors and co-activators, such as C/EBPβ and CBP/p300, as well as co-repressors [46–48]. Interestingly, SKI was found to regulate the transactivation activity of MYB as component of a HDAC-recruiting co-repressor complex together with TIF1beta, N-COR/SMRT and mSIN3A [19, 49]. Besides *MYC*, the identity of MYB target genes, for which this repressive activity of SKI might apply to, have not been characterized. Otherwise, SKI might also facilitate *MYB* expression and function, as TGFβ signaling, which is known to be repressed by SKI, inhibits *MYB* mRNA expression and hence could be counter-regulated by SKI [50, 51]. Thereby, SKI might also have an impact on MYB activity in the regulation of T and B lymphocytes [14, 24, 52–54].

*MYB* and *SKI* both are overexpressed or mutated in different tumors and leukemia subtypes [30, 46, 47]. In case of certain AML subtypes, such as AML with -7/del7q, upregulation of *SKI* expression occurs due to loss of miR-29a and therefore independent of MYB [31]. Here, we identify another potential cause of *SKI* upregulation in AML, where MYB occupies the regulatory regions in the *SKI* gene and directly induces *SKI* transcription. In AML *MYB* is well known to be deregulated, which occurs frequently via indirect mechanisms, for example by oncogenic MLL-fusion proteins or mutated SETBP1 that lead to upregulation of *MYB* expression. These perturbations result in a MYB-dependent aberrant transcriptional program and have been found to be essential in such AML for transformation and disease maintenance [55–57]. In this regard, full-length c-MYB is not able to induce leukemia in mice [58] but is essential for AML maintenance in a mouse model [55]. In contrast, overexpression of *SKI* results in a chronic neutrophilic leukemia (CNL)-resembling myeloproliferative disease in mice [59]. Further experiments are needed to analyze, if SKI is also implicated in MYB's ability to maintain AML.

**Figure 5:**
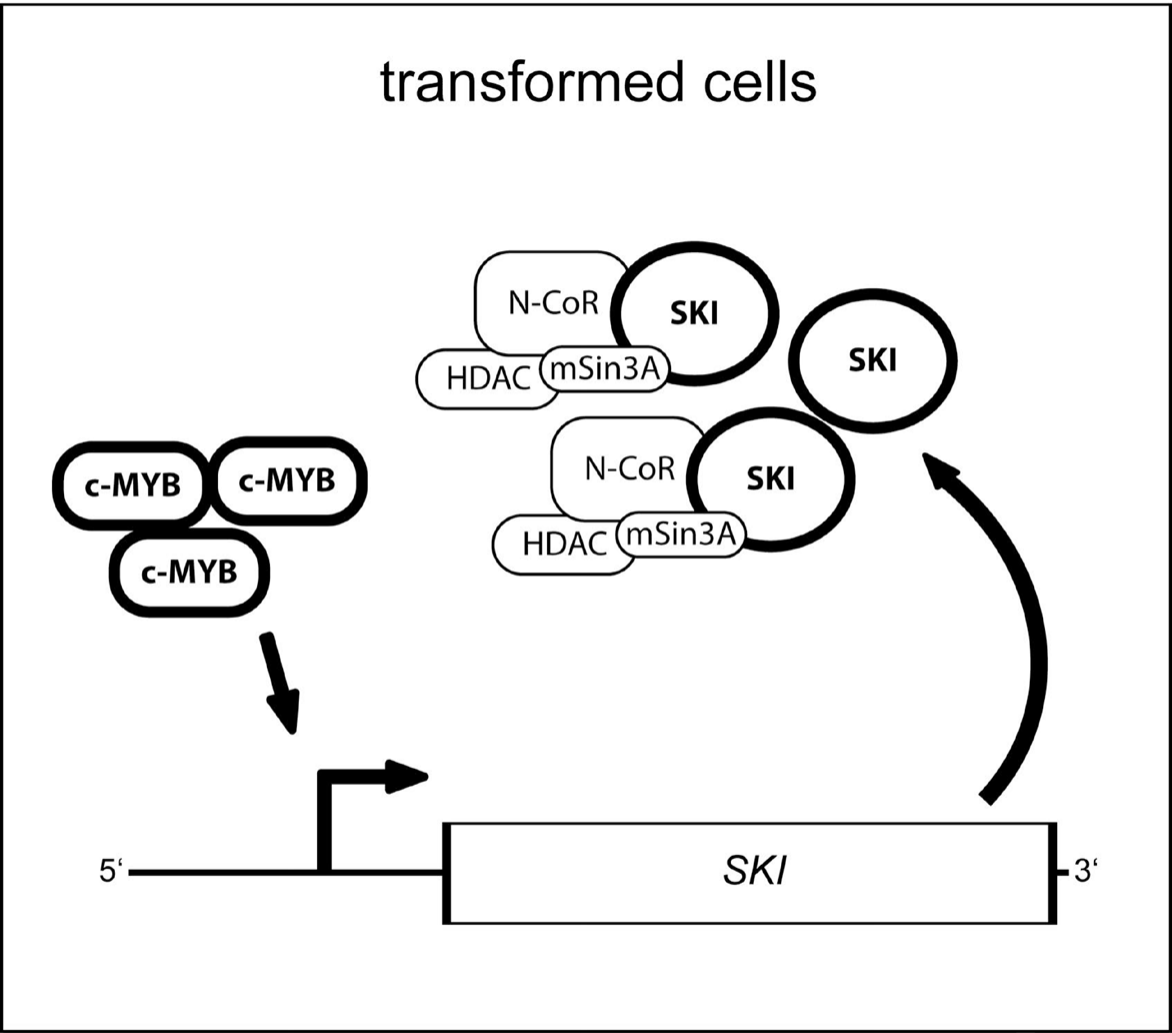
Model for the function of the MYB transcription factor and its target gene *SKI* in oncogenic cells In transformed cells deregulated and oncogenic MYB activity leads via binding to its consensus sites in the *SKI* regulatory region to upregulated expression of *SKI*. Thus, SKI protein level increases and co-repressor complex may integrate more SKI protein but, however, fails to inhibit oncogenic MYB activity as reported before by Nomura and colleagues [19] consequently enabling sustained expression of *SKI*.

Interestingly, AML cell lines such as THP1 and HL60, in which we found a good correlation of high expression levels of both *MYB* and *SKI*, reveal *MYB* deregulation and MYB dependency in their proliferation capacity [55]. Furthermore, downregulation of *MYB* in HL60 cells has been reported to induce monocytic differentiation [60]. These observations suggest that *SKI* upregulation, together with other MYB target genes, might belong to the oncogenic transcriptional response caused by deregulated *MYB* activity, as illustrated in Figure 5. In this regard, it was also reported that another MYB target gene, the transcriptional repressor *growth factor independent 1* (*GFI1*), is involved in the inhibition of monocytic differentiation [61]. Hence, our findings indicate that *SKI* is a relevant target gene of MYB in transformed cells, as it accounts, together with other MYB target genes like *GFI1*, for the MYB-dependent differentiation block. Since SKI and GFI1 are both involved in transcriptional repression, it would be interesting to analyze, if they interact and cooperate establishing the MYB-derived differentiation block.

The oncogenic capacity of MYB arises also via somatic mutations and translocations directly encompassing the MYB gene, which then lead for example to the lack of the N- or C-terminus of the MYB protein. Deletion of the N- or C-terminal domains causes the loss of co-factor binding sites, which were found also crucial for the oncogenic potential of MYB [46]. Further studies are needed to evaluate if *SKI* is also regulated by such mutated MYB and if SKI supports the activity of MYB also in this context. Various therapeutic approaches have been developed in the past, which aimed at targeting MYB, for example on the level of transcriptional elongation, via antisense RNAs, low-molecular-weight inhibitors or vaccines. However, all these strategies against MYB were so far not transferable to the patient [18, 47, 62]. *SKI* represents a novel downstream target of MYB, and serves as a promising therapeutic objective. SKI protein is part of co-repressor complexes containing chromatin-modifying activities (Figure 5), such as HDAC and PRMT5 (protein arginine methyltransferase 5). As shown in this work, both MYB and SKI levels can be decreased by the HDACi VPA and LBH589. Hence, inhibitors such as HDACi as well as the PRMT5i GSK591, might be promising strategies for the treatment of MYB- and SKI-dependent AML [31, 51, 63].

Finally, the results of this study lead us to the conclusion that *MYB* and *SKI* expression and functions are highly positively correlated in human AML. This high correlation *in vivo* and our *in vitro* studies suggest that SKI contributes to MYB oncogenic potential. Accordingly, MYB and SKI represent promising marker proteins for novel therapeutic approaches in AML.

## MATERIALS AND METHODS

### Cell culture cell lines

HL60, U937, K562, NB4, THP-1 and Kasumi-1 cells (DSMZ, Braunschweig, Germany) were cultured in RPMI1640 (10% FBS, 1% penicillin G (100 units/ml) and streptomycin (100 mg/ml)). Human embryonic kidney (HEK) 293T (DSMZ, Braunschweig, Germany) cells were cultured in DMEM (10% FBS, 1% penicillin G (100 units/ml) and streptomycin (100 mg/ml)). HL60 and U937 cells were treated with the indicated concentrations of valproic acid sodium salt (Sigma-Aldrich, Munich, Germany), LBH589 (Panobinostat) (Biomol, Hamburg, Germany) or dimethyl sulfoxide (DMSO) (Carl Roth, Karlsruhe, Germany) as solvent control. For induction of doxycycline-inducible expression of shRNAs, cell culture medium was supplemented with 1 μg/ml doxycycline (Sigma-Aldrich, Munich, Germany).

### AML patient samples and array data

Mononuclear cells were isolated from peripheral blood or bone marrow of AML patients using density gradient centrifugation. All patients provided informed consent at the University of Marburg. Patients’ characteristics are shown in Supplementary Tables 1, 2 and 3.

The samples of 17 AML patients before treatment were collected in the context of the trials AMLSG 07-04 and AMLSG 06-04. The corresponding cDNA array data have been submitted to the Gene Expression Omnibus (GEO, http://www.ncbi.nlm.nih.gov/geo/) data repository under accession no. GSE32240 [36].

### Plasmids

All shRNAs were cloned from pTRMPV [55] via EcoRI and XhoI sites into pINDUCER10-GLuc [64]. For downregulation of *MYB* the following shRNA sequences were employed: shMyb721 (=shMYB1) CAAGAAACTTGGTGTTAGGTAA and shMyb2847 (=shMYB2) ACCACCATCTTGTGTACATCTT. As non-targeting control, shRenilla was used: shRenilla (=shCtrl) CAGGAATTATAATGCTTATCTA. For overexpression of *SKI*, *SKI* cDNA was cloned via the flanking BamHI sites from pCMV-Tag2C-SKI [65] into BglII site of the pMSCV-hygro vector (Takara Bio Europe SAS, France). For transient overexpression of human HA-tagged *c-MYB*, the previously described pcDNA3-c-MYB-HA construct was used [66]. For luciferase reporter gene assays, pGL3-promoter constructs with the c-MYB binding sites of 4 x MBS2, 4 x MBS3, 4 x MBS4, 1 x MBS2 or 1 x MBS2mut enclosed in the *SKI* gene regulatory regions were generated. Synthetic oligonucleotides with four consecutive sites of MBS2, MBS3 or MBS4 or a single MBS2 unmutated or mutated were inserted in the SmaI restriction site upstream of the SV40 promoter of the pGL3-promoter vector. The pRL-TK served as normalization standard, while pGL3-control was used as negative control (all Promega, Mannheim, Germany).

### Retroviral infection

For production of lentivirus, HEK293T cells were transfected with the lentiviral expression plasmid pINDUCER10-GLuc containing shRNA against *MYB* or control and the packaging plasmids pMD2.G and psPAX2. For production of amphotropic retrovirus, PlatA packaging cells (Cell Bioloabs, San Diego, USA) were transfected with the retroviral expression plasmids pMSCV-SKI or pMSCV. Transfections were performed using Xtreme gene (Roche, Mannheim, Germany). Supernatants containing viral particles were harvested two or three days after transfection. Additionally, lentiviral supernatants were concentrated using PEG concentration. 5 × 10^5^ HL60 cells were infected with virus upon addition of polybrene (4 μg/ml) via spinocculation (1200 rpm, 37°C, 1 h). Cells were selected and maintained in the presence of 1 μg/ml puromycin (pINDUCER) or 600 μg/ml hygromycin (pMSCV).

### RNA isolation and reverse transcription quantitative PCR (RT-qPCR)

Total RNA was isolated using the RNeasy Mini Kit with on column DNase digestion (QIAGEN, Hilden, Germany). cDNA was synthesized via QuantiTect Reverse Transcription Kit (QIAGEN). RT-qPCR were carried out via the QuantiTect SYBR Green PCR Kit (QIAGEN). *MYB* and *SKI* data of the HL60 and U937 cells were normalized to endogenous *GAPDH* and were shown relative to the controls. In case of the AML patients’ samples *MYB* and *SKI* data were normalized to endogenous *GAPDH* and were shown relative to the Kasumi-1 cell line used as plate calibrator. Relative quantification was performed using the comparative Ct (2^-ΔΔCt^) method. Following RT-qPCR primers were used: GAPDH-fw: 5′-ctcctccacctttgacgctg-3′, GAPDH-rev: 5′-accaccctgttgctgtagcc-3′; SKI-fw: 5′-tctgcaccccg tgattct-3′, SKI-rev: 5′-acgtttgccgaactgaaaag-3′. In case of the AML patients’ samples SKI RT-qPCR was performed with RT^2^ qPCR Primer Assay for Human SKI (QIAGEN). For MYB RT-qPCR the QuantiTect Primer Assay Hs_MYB_1_SG (QIAGEN) was used.

### Protein isolation and Western Blot analysis

Primary antibodies used were against SKI (sc-9140; Santa Cruz Biotechnology, Dallas, USA; 1:1000), MYB (05-175; Millipore, Darmstadt, Germany; 1:1000), CDK2 (sc-163; Santa Cruz Biotechnology; 1:5000) and β-ACTIN (A1978; Sigma-Aldrich, Munich, Germany; 1:5000). Detection was performed with HRP-conjugated secondary antibodies (DAKO, Hamburg, Germany) and Amersham ECL Plus (GE Healthcare, Freiburg, Germany).

### RNA interference

HL-60 cells (2.5 × 10^6^) were transfected with 30 or 100 nM siRNA via Amaxa Nucleofector technology (Lonza, Basel, Switzerland) and harvested 24 hours after transfection. Human *MYB* siRNAs #1 - #4 or pool (D-003910-01, -02, -03, -04, M-003910-00) and non-targeting siRNAs #1 or #5 (D-001210-01, D-001210-05) were purchased from Dharmacon (GE Healthcare).

### Chromatin immunoprecipitation and quantitative PCR (ChIP-qPCR)

For chromatin immunoprecipitation (ChIP) assays HL-60 or AML patient cells were crosslinked for 10 min with 1% formaldehyde at 37°C. The reaction was stopped by addition of glycine 0.125 M for 5 min at 37°C. Cell lysis was executed in Lysis buffer I (5 mM PIPES pH 8, 85 mM KCl, 0.5% NP-40, protease inhibitors) for 20 min on ice. After centrifugation cells were resuspended in Lysis buffer II (10 mM Tris/HCl pH 7.5, 150 mM NaCl, 1% NP-40, 1% sodium deoxycholate, 0.3% SDS, 1 mM EDTA, protease inhibitors) and incubated on ice for 10 min. Chromatin was fragmented by sonification (50 × 3 s on ice at 25% amplitude, Branson Sonifier W-250-D). For immunoprecipitations (IP), chromatin was precleared with Protein A Sepharose (GE Healthcare, Freiburg, Germany) for 2 h, which had been incubated overnight with 1 mg/ml BSA and 400 μg/ml salmon sperm DNA. IPs were performed with 4 μg of the following antibodies overnight at 4°C: MYB (mouse; clone 1-1; 05-175; Millipore, Darmstadt, Germany), and mouse IgG (Sigma-Aldrich, Munich, Germany). 50% beads slurry was added and incubated for 2 h at 4°C. Beads were washed twice with washing buffer I (20 mM Tris/HCl pH 8.1, 150 mM NaCl, 2 mM EDTA, 0.1% SDS, 1% Triton X-100), washing buffer II (20 mM Tris/HCl pH 8.1, 500 mM NaCl, 2 mM EDTA, 0.1% SDS, 1% Triton X-100), washing buffer III (10 mM Tris/HCl pH 8.1, 250 mM LiCl, 1 mM EDTA, 0.1% sodium deoxycholate, 1% NP-40) and TE buffer. Chromatin was eluted with 0.1 M NaHCO_3_ and 1% SDS for 15 min at RT and crosslinking was reversed via incubation for 3 h at 55°C and after addition of Proteinase K at 65°C overnight. Chromatin was purified via QIAquick columns (QIAGEN, Hilden, Germany) and analyzed using Absolute qPCR SYBR Green Mix (Thermo Scientific) with the following primers for the c-MYB binding sites (MBS) MBS1, MBS2, MBS3 and MBS4: MBS1-fw: 5′-ctggcctgaatgctcttttc-3′, MBS1-rev: 5′-ttcctggcaagaaaaccac-3′; MBS2-fw: 5′-actgccgc ctcatgtacc-3′, MBS2-rev; 5′-tcctccttgcccgtgtaat-3′, MBS3-fw: 5′-agctgcagacagacgtagca-3′, MBS3-rev: 5′-aa ccaactccccgtgtga-3′; MBS4-fw: 5′-tggggaatgactgagcttg-3′, MBS4-rev; 5′-aaacacaactcctccccaga-3′. A further downstream region within the *SKI* gene served as control: ds ctrl-fw: 5′-gggcactaactgggttcttg-3′, ds ctrl-rev: 5′-cacacaagctgtcgtggagt-3′. MYC served as a positive control: MYC-fw: 5′-aaaaggggaaagaggacctgg-3′, MYC-rev: 5′-cctaaaaggggcaagtggagag-3′. ChIP-qPCR results were expressed as % input or fold IgG. Each ChIP-qPCR reaction was performed in triplicates from the same experiment (technical replicates) and the standard deviation (indicated by error bars) was calculated accordingly. The presented HL60 data sets are representative of at least 3 independent experiments (biological replicates). The AML patient data sets are representative of 2 independent experiments (biological replicates).

### Luciferase reporter assay

For luciferase reporter gene assays, HEK293T cells were co-transfected with pGL3-promoter-4 × MBS2 - 4 × MBS4, -1 × MBS2 or -1 × MBS2mut constructs or pGL3-control (Promega, Mannheim, Germany), pRL-TK (Promega) and pcDNA3-empty or pcDNA3-c-MYB. After 48 h luciferase activity was measured using the Dual Luciferase Reporter Assay System (Promega). Firefly luciferase (pGL3) activity was normalized to renilla luciferase (pRL-TK) activity or total protein concentration. Total protein concentration of each sample was determined by bicinchoninic acid assay (BCA assay) with BSA standard curve. The results were depicted relative to mock transfected cells.

### Flow cytometry

HL60 cells were stimulated ± doxycycline for 48 h. Flow cytometric analyses were consecutively performed on 1 × 10^6^ cells stained with a phycoerythrin-labeled anti-human CD11b antibody (Beckman Coulter, Krefeld, Germany). To exclude death cells, cells were additionally stained with DAPI (4’,6-diamidino-2-phenylindole).

### Statistical analysis

Data are presented as means ± s.d. for at least three independent experiments. Correlations among protein or transcript levels of *MYB* and *SKI* were examined by Pearson's correlation. Significance was determined using two-tailed Student's *t*-test and was defined as *P* < 0.05.

## SUPPLEMENTARY MATERIALS FIGURES AND TABLES




