# Supplementary material for: MYB induces the expression of the oncogenic corepressor SKI in acute myeloid leukemia
Source: Oncotarget. 2018 Apr 27;9(32):22423–35. doi: 10.18632/oncotarget.25051 (PMC5976475; doi:10.18632/oncotarget.25051)
Supplement: Supplementary file 1 [file oncotarget-09-22423-s001.pdf]

MYB induces the expression of the oncogenic corepressor SKI in acute myeloid leukemia

SUPPLEMENTARY MATERIALS

Plasmids

For luciferase reporter gene assays, pGL3-promoter constructs with the c-MYB binding sites of 4 × MBS2 or 4 × MBS2mut enclosed in the *SKI* gene regulatory regions were generated. Synthetic oligonucleotides with four consecutive sites of MBS2 unmutated or mutated were inserted in the SmaI restriction site upstream of the SV40 promoter of the pGL3-promoter vector.

ChIPseq analyses

We obtained raw fastq files from the gene expression omnibus (GEO) database under the accession number GSE59657 [1]. Raw reads were aligned with Bowtie2 (v.2.0.0-beta7) [2] to a human reference genome from the ensembl data base, revision 86. Subsequently, aligned reads were deduplicated to the expected number of duplicate reads based on a binomial distribution estimate. The effective reads were then used as input for the peak caller MACS (v.1.4.0rc2) [3], which was used to identify enriched "peak" regions within the alignments. Regions containing peaks of interest were visualized using the IGV genome browser (v.2.3).

Luciferase reporter assay

For luciferase reporter gene assays, HEK293T cells were co-transfected with pGL3-promotor-4 × MBS2 or 4 × MBS2mut constructs and pcDNA3-empty or pcDNA3-c-MYB. After 48 h luciferase activity was measured using the Dual Luciferase Reporter Assay System (Promega). Firefly luciferase (pGL3) activity was normalized to total protein concentration. Total protein concentration of each sample was determined by bicinchoninic acid assay (BCA assay) with BSA standard curve. The results were depicted relative to mock transfected cells.

RNA interference and overexpression experiments

For knockdown of MYB, U937 cells (1 × 10^6) were transfected with 100 nM siRNA via Amaxa Nucleofector technology (Lonza, Basel, Switzerland) and harvested 24 h after transfection. Human *MYB* siRNApool (M-003910-00) and non-targeting siRNA #5 (D-001210-05) were purchased from Dharmacon (GE Healthcare). For overexpression of MYB, K562 cells (1 × 10^6) were transfected with 3 µg pCDNA3 (Invitrogen by Thermo Fisher Scientific) or pcDNA3-c-MYB-HA [4] via Amaxa Nucleofector technology (Lonza, Basel, Switzerland) and harvested 48 h after transfection.

REFERENCES

1. Mansour MR, Abraham BJ, Anders L, Berezovskaya A, Gutierrez A, Durbin AD, Etchin J, Lawton L, Sallan SE, Silverman LB, Loh ML, Hunger SP, Sanda T, et al. Oncogene regulation. An oncogenic super-enhancer formed through somatic mutation of a noncoding intergenic element. Science. 2014; 346:1373–1377.
2. Langmead B, Salzberg SL. Fast gapped-read alignment with Bowtie 2. Nature Methods. 2012; 9:357–359.
3. Zhang Y, Liu T, Meyer CA, Eeckhoute J, Johnson DS, Bernstein BE, Nusbaum C, Myers RM, Brown M, Li W, Liu XS. Model-based analysis of ChIP-Seq (MACS). Genome Biol. 2008; 9:R137.
4. Zhao H, Jin S, Gewirtz AM. The histone acetyltransferase TIP60 interacts with c-Myb and inactivates its transcriptional activity in human leukemia. J Biol Chem. 2012; 287:925–934.

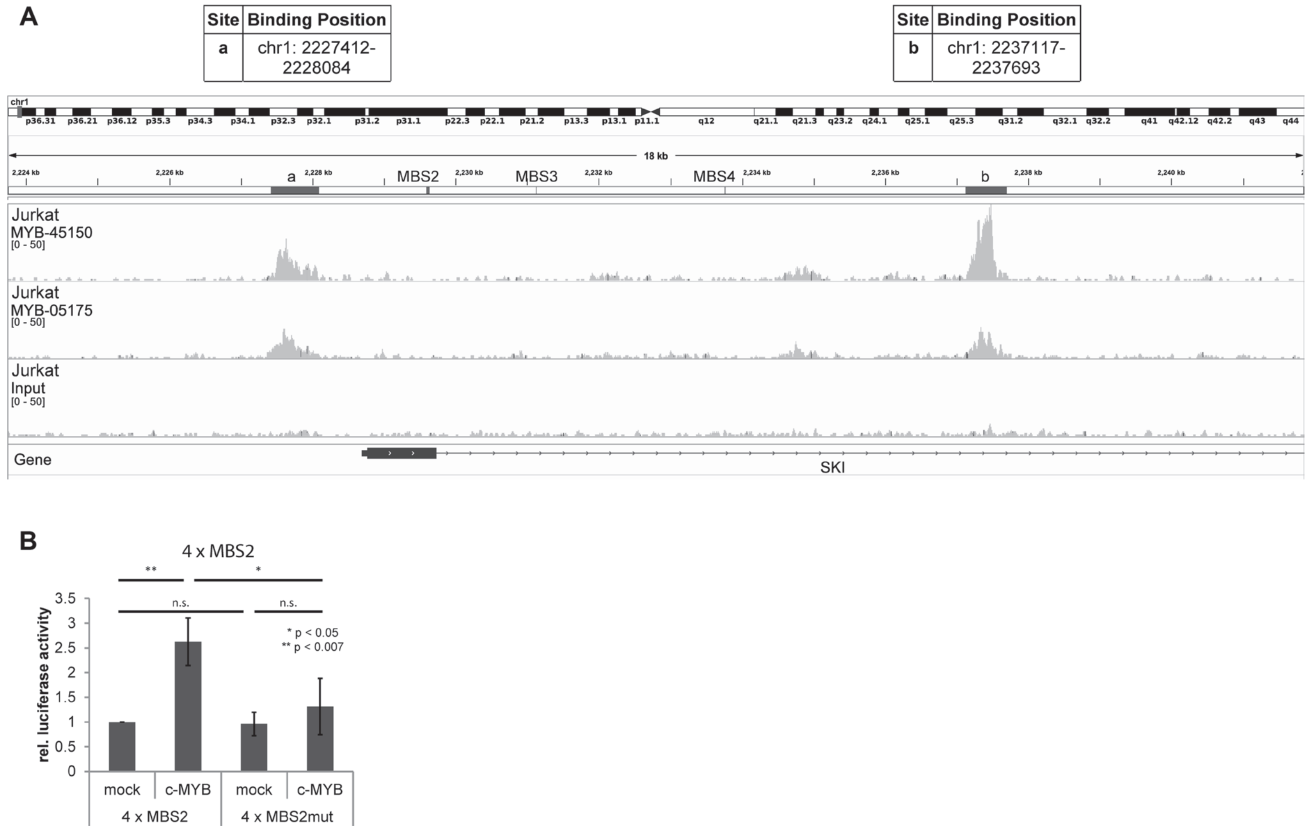

Supplementary Figure 1: ChIPseq analysis and reporter gene assays regarding the transcriptional regulation of the human *SKI* gene by MYB. (**A**) ChIPseq tracks at the SKI locus from Jurkat T-acute lymphoblastic leukemia (T-ALL) cells for MYB (anti-MYB: Abcam, ab45150 and Millipore, 05–175) and input of the dataset of Mansour *et al*. [1]. The two MYB lymphoid interaction sites a and b as well as the myeloid interaction sites MBS2, MB3 and MBS4 are depicted as grey boxes. The binding positions in chromosome 1 (chr1) of a and b refer to the human reference genome hg38 (GRCh38). (**B**) HEK293T were co-transfected with MYB-HA or empty vector (mock), pGL3-promoter-4 × MBS2 or -4 × MBS2mut. The data ($n = 4$, mean ± s.d.) were normalized to total protein concentration. Construct pGL3-promoter-4 × MBS2 co-transfected with the empty vector (mock) was set to 1. $^{*}P < 0.05$, $^{**}P < 0.007$.

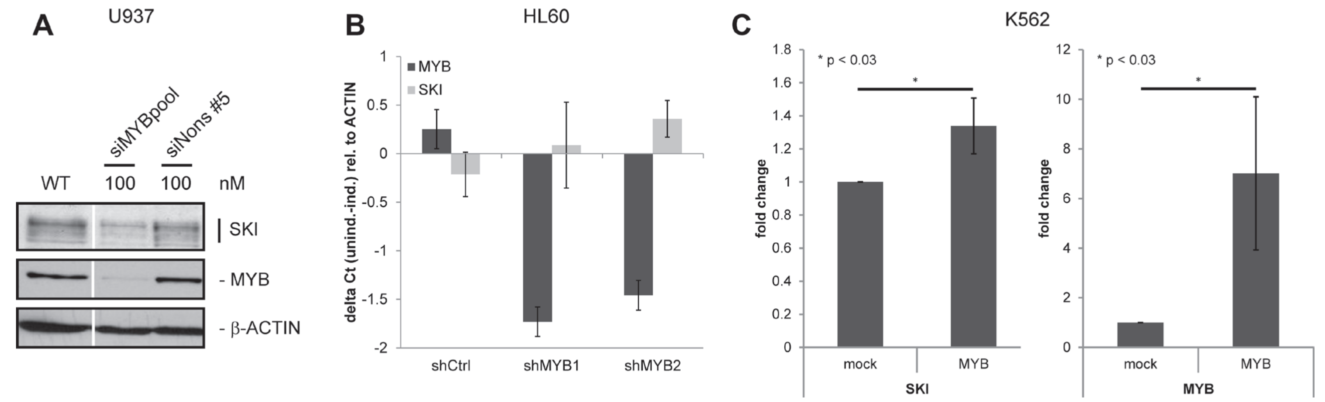

Supplementary Figure 2: *SKI* expression is positively regulated by MYB in leukemic cell lines. (**A**) Western Blot analysis for SKI and MYB in U937 WT or transfected with indicated amounts of siMYBpool or siNons #5. Cells were harvested 24 h after transfection. β-ACTIN served as loading control. (**B**) RT-qPCR analysis for *MYB* and *SKI* transcripts in HL60 cells expressing shRNAs against MYB or a control shRNA after 48 h doxycycline treatment. (**C**) RT-qPCR analysis of four independent experiments performed in duplicates for *MYB* and *SKI* transcripts in K562 cells transfected with pCDNA3-empty (mock) or pCDNA3-HA-MYB (MYB). Cells were harvested 48 h after transfection. Values are normalized to *GAPDH* and plotted relative to mock (mean ± s.d.). $^{*}P < 0.03$.

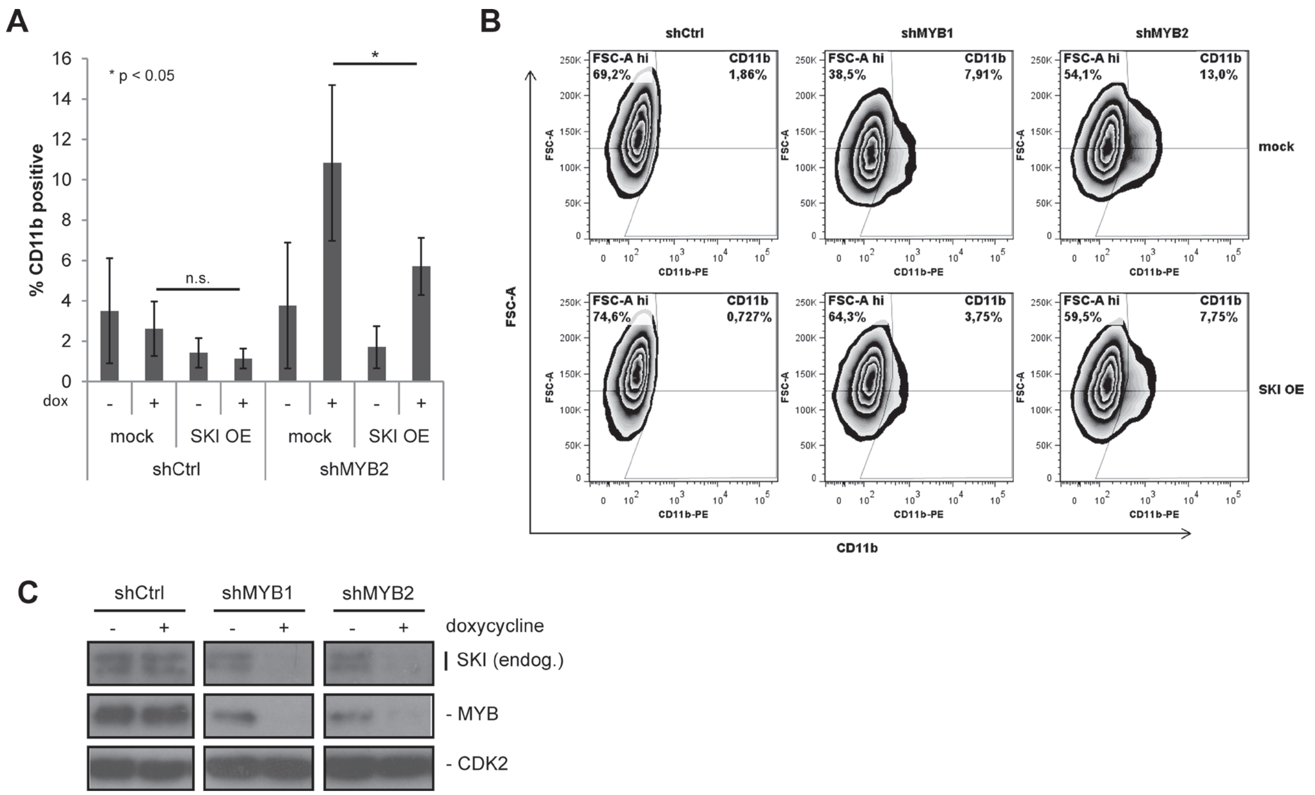

Supplementary Figure 3: *SKI* overexpression attenuates MYB dependent differentiation and SKI expression is decreased upon down-regulation of MYB in AML cells. (**A**) Expression of CD11b was analyzed by FACS in HL60 expressing a shRNA (shMYB2) targeting *MYB* or a control shRNA (shCtrl) in absence or presence of *SKI* overexpression (SKI OE). The empty vector (mock) served as negative control for *SKI* overexpression. Expression of shRNAs was induced by doxycycline (dox) and cells were measured via FACS (anti-human CD11b-PE) 48 h later. Bar graphs show mean values ± s.d. of % CD11b-positive HL60 of four independent experiments. $^{*}P < 0.05$. (**B**) One typical flow cytometrical analysis from four independent experiments of doxycycline-induced HL60 cells showing changes in cell differentiation by means of cell size (FSC-A) and differentiation marker CD11b. The experiment was performed and measured as described in (a). (**C**) Western Blot analysis for endogenous SKI and MYB in HL60 cells expressing alternative shRNAs (shMYB1, shMYB2) targeting MYB or a control shRNA (shCtrl) upon doxycycline induction. CDK2 served as a loading control.

Supplementary Table 1: Characteristics of AML patients used for Western Blot analyses. See Supplementary_Table_1

Supplementary Table 2: Characteristics of AML patients used for RT-qPCR analyses

No.	gender	age (years)	material	blasts (%)	FAB	FLT3-ITD mutation	NPM1 mutation	other mutations	karyotype
1	m	69	BM	35	sAML M2 from MDS				47, XY, +11 [8], 47, XY, +8 [3], 46, XY [9]
2	f	23	BM	30–50	M4/M5	NE	NE	NE	46, XX, t(9;22) (q34;q11) [7] 45, XX, −7, t(9;22) (q34;q11) [10] 46, XX [3]
3	m	64	BM	30	M4	x			46,XY [20]
4	m	48	pB	80	M4/Eo				46, XY [6]; 46, XY, inv. (16) (p13q22) [18]
5	m	48	BM	60	sAML from RAEBII				47, XY, t(3,21) (q26;q11), +der(21) t(3;21)(q26;q11) [20]
6	m	55	pB	30	M4	NE	NE	NE	complex
7	f	57	BM	80	M2				46, XX [20]
8	f	33	pB	90	M5a				46, XX. der(19) ins(19;11) (p13;q23q23) (MLL5+) [20]
9	f	56	BM	30	M2		x		47 XX, +4 [4]; 46 XX [20]
10	m	71	pB	54	sAML				45 XY, −7 [4]/46XY, −7, +mar [19]/47, XY, −7, +8, +mar [2]
11	m	44	pB	64	M5a		x		46, XY
12	m	74	BM	50	M4				45, X, −Y[20]
13	f	30	BM	31	M4	x	x		46, XX
14	f	46	BM	24	M2				d(5q)
15	f	64	BM	91	M4	x		MLL-PTD	47, XX, +ider(11) del(11)(q11q23) [20]
16	m	50	BM	50	M4			FLT3-TKD	46 XY t(3; 5) (q25; q34) [20], 46 XY [1]
17	f	71	BM	50	M5	x			46XY [20]
18	m	77	BM	76	sAML M4 from RAEBII				46, XY, t(2;9) (p13;q22) [20]
19	f	48	BM	40	M4 with mulitlineage dysplasia	x		MLL-PTD, RUNX1	46, XX [20]
20	f	48	BM	26	M4			RUNX1	46, XX [20]
21	m	70	BM	98	M2	x	x		NE

BM, bone marrow; NE, not evaluated; pB, peripheral blood; sAML, secondary AML.

Supplementary Table 3: Characteristics of AML patient used for ChIP-qPCR analysis

No.	gender	age (years)	material	blasts (%)	FAB	FLT3-ITD mutation	NPM1 mutation	other mutations	karyotype
1	m	61	pB	98	NE	x	x	DNMT3A mut, IDH1 mut	46, XY [20]

pB, peripheral blood; NE, not evaluated.
