# Supplementary material for: MYB induces the expression of the oncogenic corepressor SKI in acute myeloid leukemia
Source: Oncotarget. 2018 Apr 27;9(32):22423–35. doi: 10.18632/oncotarget.25051 (PMC5976475; doi:10.18632/oncotarget.25051)
Supplement: Supplementary file 2 [file oncotarget-09-22423-s002.pdf]

Supplementary Table 1: Characteristics of AML patients used for Western Blot analyses

No.	gender	age (years)	material	blasts (%)	FAB	FLT3-ITD mutation	NPM1 mutation	other mutations	karyotype
1	f	35	BM	90	M4			C/EBPA mut	46XX
2	m	66	pB	83	M0				46,XY,del(7)(q11) [20]
3	m	50	pB	70	M2	x	x	FLT3-TKD	46,XY
4	m	77	BM	76	M4				46, XY, t(2;9)(p13;q22) [20]
5	m	75	BM	83	M5	x			46XY[20]
6	m	69	BM	35	sAML M2 from MDS				47, XY, +11 [8], 47, XY, +8 [3], 46, XY [9]
7	f	74	BM	76	M4				46 XX t(8;21)(q22;q22) (t (8;21); 47 XX +4, t(8;21)(q22;q22)
8	f	23	BM	30-50	M4/M5	NE	NE	NE	46, XX, t(9;22)(q34;q11)[7] 45, XX, -7, t(9;22)(q34;q11)[10] 46, XX [3]
9	m	47	BM	30	M4		x		46, XY [20]
10	f	75	pB	98	M3				t(15;17)
11	m	53	pB	20	sAML			N-RAS mut	46,XY[20]
12	f	75	BM	40-60	M4		x	FLT3-TKD	47,XX, +4 [7] 46,XX [13]
13	f	51	BM	60	M4	x	x		46, XX [20]
14	f	59	BM	89	NE	x	x		46, XX [14]
15	f	66	BM	91	M5				47, XX, r(7)(p15q21),+i(11)(q10) [19]46, XX
16	m	47	pB	88	M2	x	x		46, XY [20]
17	f	80	pB	94	M2	NE	NE	NE	NE
18	f	73	pB	40	M4				46,XX [20]
19	f	53	pB	BM 80	M4		x		46, XX [27]
20	f	24	BM	71	M4		x		46, XX [25]
21	f	63	BM	20-30	M4				46,XX, del(5)(q14;q34)[19]
22	f	58	pB	20	M0/M1			NF1 del	46,XX,deletion(11)(p12p14),del(17)(p11p12). ish der(17)del(17)(p11p12)del(17)(q11q11)(pt53 +,ns1-)[18]46,XX[2]
23	f	56	pB	70	M4		x		48,XX,+8+8[8], 46,XX[12]

24	m	66	pB	BM 85	M2			FLT3-TKD, MLL-PTD	46 XY [20]
25	f	78	pB	35	M5			CBFB del, TP53 del	44,XX,del(5)(q14q34), dic(7;22)(q11;p11),+8,der(13;16)(q10p10),dic (17;18)(p12;p11) [11]45, idem, +9 [2] (MLL)
26	m	64	pB	24	M1/M2			MLL-PTD	46,XY [20]
27	f	61	BM	93	M5	x	x		46,XX,t(4;6) (q11;q27) [19], 46,XX [2])
28	m		pB	83	M4			MLL-PTD	NE
29	m	49	BM	30-40	sAML from RAEBII				46,XY [20]
30	m	62	pB	BM 80-90	M4		x		46,XY -> 20-30% Promyelozyten pB
31	m	73	BM	90	M2		x		46,XY [20]
32	m	73	pB	91	M5		x		46,XY [20]
33	f	75	BM	30	M4		x	MLL-PTD	46,XX,+13,-21 [8]
34	f	34	BM	90	M4			MLL-AF6	46, XX, t(6:11)q(27;q23) [19]
35	m	80	pB	80	M2		x	FLT3-TKD	46 XY [20]

ABBREVIATIONS: BM, bone marrow; NE, not evaluated; pB, peripheral blood; sAML, secondary AML.
